# Effects of Turmeric and Turmeric Plus Piperine Supplementation on Lipid Profiles in Adults with Cardiometabolic Risk Conditions: A Systematic Review and Meta-Analysis of Randomized Controlled Trials Following PRISMA Guidelines

**DOI:** 10.3390/pharmaceutics17121609

**Published:** 2025-12-15

**Authors:** Francisco Epelde

**Affiliations:** Internal Medicine Department, Institut d’Investigació i Innovació Parc Taulí (I3PT-CERCA), Parc Taulí Hospital Universitari, Universitat Autònoma de Barcelona, 08208 Sabadell, Spain; fepelde@gmail.com

**Keywords:** turmeric, curcumin, piperine, lipid profile, dyslipidemia, nutraceuticals, meta-analysis, PRISMA

## Abstract

**Background**: Turmeric (*Curcuma longa*) and its main bioactive compound curcumin are widely promoted for cardiometabolic health, yet their efficacy on lipid parameters remains uncertain. Piperine, an alkaloid from black pepper, enhances curcumin bioavailability and may potentiate its effects. This systematic review and meta-analysis aimed to assess the impact of turmeric, alone or combined with piperine, on lipid profiles in adults with metabolic disorders. **Methods**: A systematic search was conducted (2010–2025) in PubMed, Scopus, and Cochrane CENTRAL. Randomized controlled trials (RCTs) evaluating turmeric supplementation (with or without piperine) on lipid outcomes were included. Methodological quality was assessed with Cochrane RoB 2; certainty of evidence was rated using GRADE. Meta-analyses were performed with random-effects models. The protocol followed PRISMA 2020 guidelines. **Results**: Ten records were identified; six full texts were assessed; three RCTs (n ≈ 250) were included in quantitative synthesis, and three additional RCTs narratively. Pooled analysis demonstrated significant reductions in triglycerides (WMD −25.5 mg/dL, 95% CI −32.5 to −18.4), total cholesterol (−14.1 mg/dL, 95% CI −22.9 to −5.3), and LDL-C (−17.0 mg/dL, 95% CI −25.2 to −8.8), with an increase in HDL-C (+5.7 mg/dL, 95% CI +2.0 to +9.4). Subgroup analysis suggested greater LDL-C reduction with turmeric+piperine (−29.6 mg/dL) compared to turmeric alone (−16.2 mg/dL). Certainty of evidence was moderate for TG, TC, LDL-C, and low for HDL-C. **Conclusions**: Turmeric supplementation, particularly when combined with piperine, improves lipid profiles in adults with metabolic disorders. These effects are clinically relevant and comparable to other nutraceuticals, although evidence remains limited by short trial duration and small sample sizes. Larger, long-term RCTs are warranted before turmeric can be recommended in evidence-based dyslipidemia guidelines.

## 1. Introduction

Cardiovascular disease (CVD) is the leading cause of morbidity and mortality globally. Dyslipidemia—characterized by elevated serum triglycerides (TG), total cholesterol (TC), low-density lipoprotein cholesterol (LDL-C), and decreased high-density lipoprotein cholesterol (HDL-C)—is one of the most important modifiable risk factors for atherosclerosis and ischemic heart disease [1]. Despite effective lipid-lowering pharmacotherapies such as statins, ezetimibe, and PCSK9 inhibitors, a substantial residual cardiovascular risk persists [2]. Moreover, issues of drug intolerance, limited accessibility, and patient preference for “natural” alternatives contribute to the increasing demand for nutraceuticals and complementary therapies [3,4,5].

Turmeric (*Curcuma longa*), a yellow spice widely used in traditional Asian medicine and cuisine, has been promoted extensively in recent years as a natural agent for cardiovascular protection [6]. Its principal bioactive compounds, curcuminoids (curcumin, demethoxycurcumin, and bisdemethoxycurcumin), have demonstrated antioxidant, anti-inflammatory, and metabolic regulatory effects in preclinical models [7]. These actions include suppression of lipogenesis, activation of LDL receptors, promotion of cholesterol efflux, and reduction in oxidative stress—all mechanisms potentially relevant to lipid modulation and atherosclerosis prevention [8].

Given these properties, turmeric and curcumin-based supplements are now widely marketed and recommended in commercial and wellness settings as lipid-lowering agents. They are commonly advertised as “natural cholesterol regulators” or “cardiovascular protectors,” often highlighting their traditional use and safety profile. Such claims have contributed to widespread consumer use, frequently without direct supervision by healthcare providers [9]. However, despite their popularity, the actual clinical efficacy of turmeric and curcumin in improving serum lipids remains uncertain.

A critical limitation is that oral curcumin exhibits poor systemic bioavailability due to rapid intestinal and hepatic metabolism. To overcome this, formulations combining curcumin with piperine, an alkaloid extracted from black pepper (*Piper nigrum*), have been developed. Piperine inhibits glucuronidation and efflux transporters, thereby enhancing curcumin plasma concentrations by up to 2000% [10]. Moreover, piperine itself has been suggested to exert metabolic effects, including improved insulin sensitivity and modulation of peroxisome proliferator-activated receptors (PPARs), which may contribute to lipid regulation independently of curcumin.

The scientific gap. Despite growing commercial promotion and consumer adoption, robust clinical evidence remains limited. Randomized controlled trials (RCTs) investigating turmeric, curcumin, and curcumin–piperine combinations have produced inconsistent findings [11]. Some studies report clinically meaningful reductions in LDL-C (>20 mg/dL) and triglycerides, while others show neutral or modest results. Variability in study populations (metabolic syndrome, type 2 diabetes mellitus, non-alcoholic fatty liver disease [NAFLD]) [12], intervention duration, formulations [13] (standard extract, nano-curcumin, phytosomal), and methodological quality contribute to these discrepancies [14,15].

Previous systematic reviews and meta-analyses [16,17,18,19,20,21,22,23,24,25,26,27,28,29,30,31,32,33] concluded that curcumin supplementation had little to no effect on lipid parameters [17]. More recent umbrella reviews and narrative syntheses suggest possible benefits, but with substantial heterogeneity, incomplete reporting, and low certainty of evidence [18,19,20]. Importantly, most prior reviews did not systematically distinguish between turmeric alone and turmeric combined with piperine, nor did they consistently apply modern methodological standards such as PRISMA 2020, the Cochrane risk-of-bias (RoB 2) tool, or GRADE certainty assessment [21,22,23,24,25,26,27,28,29].

Rationale and objectives. In this context, there is a pressing need for a methodologically rigorous evaluation of the lipid-modifying effects of turmeric and its combinations with piperine, beyond marketing claims and anecdotal evidence. Only through systematic synthesis of randomized controlled trials can the clinical efficacy and limitations of these supplements be clarified. Such evidence is essential to determine whether turmeric-based nutraceuticals can be responsibly recommended as adjunctive therapies in dyslipidemia management. Given the growing use of turmeric-based supplements in individuals with cardiometabolic risk—and emerging evidence suggesting potential benefits not only on lipid profiles but also on glycemic control, body weight regulation, low-grade inflammation, and neurocognitive health—a rigorous synthesis of randomized controlled trials specifically focused on lipid outcomes is warranted to clarify its role in cardiometabolic prevention.

Therefore, the objectives of this systematic review and meta-analysis were as follows:

1. Quantify the effects of turmeric and turmeric + piperine supplementation on TG, TC, LDL-C, and HDL-C in adults with metabolic disorders;
2. Evaluate differences between turmeric alone and turmeric combined with piperine;
3. Assess methodological quality, risk of bias, and certainty of evidence;
4. Integrate non-meta-analyzable but relevant RCTs into a structured narrative synthesis;
5. Provide an evidence-based perspective to guide clinicians, researchers, and policy-makers on the role of turmeric supplementation in lipid management.

## 2. Materials and Methods

### 2.1. Protocol and Reporting

This systematic review and meta-analysis were conducted in accordance with the PRISMA 2020 statement (Supplementary Materials) and Cochrane methodological standards. A review protocol was developed a priori and submitted for prospective registration in PROSPERO, the International Prospective Register of Systematic Reviews (Registration ID: CRD1242256). All stages of the process—including study identification, screening, eligibility, extraction, and synthesis—were conducted by two independent reviewers, with discrepancies resolved by consensus.

### 2.2. Eligibility Criteria

Study design: Randomized controlled trials (parallel or crossover).

Population: Adults (≥18 years) with dyslipidemia, metabolic syndrome, obesity, type 2 diabetes mellitus (T2DM), or non-alcoholic fatty liver disease (NAFLD).

Intervention: Oral turmeric, curcumin, or curcuminoid formulations, administered alone or in combination with piperine. Minimum intervention duration was 8 weeks.

Comparator: Placebo, no treatment, or standard of care.

Outcomes: Serum lipid profile: TG, TC, LDL-C, and HDL-C. Trials reporting quantitative changes with dispersion measures (SD, SEM, or 95% CI) were eligible for pooling.

Exclusion criteria:

- Observational or non-randomized studies
- Pediatric populations
- Duration < 8 weeks
- Multi-component nutraceuticals in which turmeric/curcumin was combined with other bioactive botanicals or nutraceutical ingredients (e.g., polyherbal formulas), with the exception of predefined curcumin + piperine combinations, which were eligible as a mechanistically justified enhancer of curcumin bioavailability.
- Trials lacking extractable dispersion measures
- Lipoprotein(a) [Lp(a)] was considered an outcome of interest; however, none of the eligible RCTs reported Lp(a) values suitable for quantitative synthesis.

### 2.3. Information Sources and Search Strategy

We searched PubMed, PubMed Central (PMC), and publisher repositories from inception through 1 July 2025 using the following Boolean strategy:

(curcumin OR turmeric OR curcuminoids) and

(lipid* OR cholesterol OR triglycerid* OR LDL OR HDL) and

(random* OR placebo OR trial)

Reference lists of eligible articles and prior reviews were hand-searched to ensure completeness.

### 2.4. Study Selection

After removal of duplicates, titles and abstracts were screened. Full texts were retrieved if potentially relevant. Studies were included if they met all eligibility criteria. Reasons for exclusion were recorded at the full-text stage. During full-text screening, RCTs evaluating turmeric as part of broad multi-ingredient formulations were excluded to avoid confounding and to ensure that effect estimates reflected turmeric or turmeric+piperine specifically.

**Figure 1:**
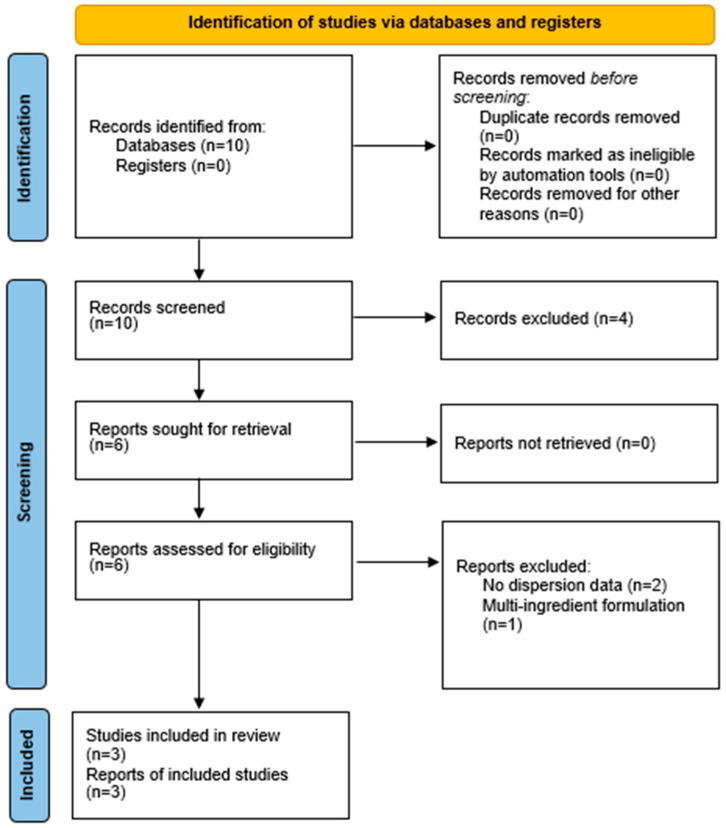
PRISMA 2020 flow diagram.

The process is illustrated in the PRISMA 2020 flow diagram (Figure 1).

### 2.5. Data Collection Process

Two reviewers independently extracted data into a standardized form:

- Study details (author, year, country, sample size, condition)
- Intervention characteristics (formulation, dose, duration, co-supplementation with piperine)
- Comparator details
- Lipid outcomes (mean change ± SD/SEM, 95% CI, or calculated differences)
- Funding source and conflicts of interest

When results were reported only as confidence intervals or within-group changes, we derived between-group mean differences and standard errors using error propagation formulas. Because of heterogeneous formulations (standardized extracts, nano-curcumin, and curcuminoids with piperine) and incomplete pharmacokinetic reporting, we did not mathematically standardize interventions to a single ‘curcumin-equivalent’ dose. Instead, we restricted inclusion to trials with clearly defined turmeric/curcumin-based regimens and prespecified subgroup analyses by formulation (turmeric alone vs. turmeric + piperine).

### 2.6. Risk of Bias in Individual Studies

The Cochrane Risk of Bias 2 (RoB 2) tool was applied independently by two reviewers across five domains: randomization, deviations from intended interventions, missing data, measurement of outcomes, and selective reporting. Overall risk was judged as low, some concerns, or high. The included RCTs employed different formulations—standardized curcumin extracts, curcuminoid mixtures combined with piperine, and nano-curcumin preparations—at daily doses ranging from 80 mg (nano-curcumin) to 1890 mg (conventional extract). It is well established that native curcumin has poor oral bioavailability, while co-administration with piperine and advanced delivery systems (e.g., nanoformulations, phospholipid complexes) substantially increases systemic exposure. Consequently, nominal dose alone is not directly comparable across formulations, and observed lipid effects likely reflect both dose and formulation-dependent bioavailability rather than milligram dose per se.

### 2.7. Effect Measures and Synthesis

Outcomes were expressed as weighted mean differences (WMD, mg/dL) between intervention and control.

We used random-effects models (DerSimonian–Laird), given expected clinical heterogeneity. As a sensitivity analysis, all primary outcomes were additionally analyzed using fixed-effect models; these yielded effect estimates closely overlapping with those of the random-effects models, without changing the direction or statistical significance of any lipid parameter. Heterogeneity was assessed using the I^2^ statistic, with thresholds of 25%, 50%, and 75% for low, moderate, and high inconsistency.

Subgroup analyses were performed comparing

- Turmeric alone (standard extract, nano-curcumin, phytosome)
- Turmeric + piperine

### 2.8. Certainty of Evidence

The GRADE framework was applied to each outcome, considering risk of bias, inconsistency, indirectness, imprecision, and publication bias. Certainty was rated as high, moderate, low, or very low.

This diagram illustrates the full study selection process.

- Records identified: 10
- Full texts assessed: 6
- Excluded: 3 (reasons: no dispersion data [2]; multi-ingredient formulation [1])
- Included in qualitative synthesis: 3 + 3 narratively summarized
- Included in quantitative synthesis (meta-analysis): 3

## 3. Results

### 3.1. Study Results

The search yielded 10 records. After screening and eligibility assessment, three RCTs (*n* ≈ 250 participants) provided extractable data and were included in the quantitative meta-analysis. Three additional RCTs were included narratively but were not meta-analyzable due to incomplete reporting (e.g., absence of dispersion measures or co-interventions). Reasons for exclusion are detailed in the PRISMA 2020 flow diagram (Figure 1).

### 3.2. Study Characteristics

Table 1 summarizes the included studies, briefly described as follows:

- Yang 2014 (China): metabolic syndrome, turmeric extract 1890 mg/day vs. placebo, 12 weeks [30]
- Panahi 2014 (Iran): metabolic syndrome, curcuminoids 1000 mg/day + piperine 10 mg/day vs. placebo, 8 weeks [31].
- Jazayeri-Tehrani 2019 (Iran): NAFLD, nano-curcumin 80 mg/day vs. placebo, 12 weeks [32].

### 3.3. Risk of Bias

As shown in Table 2, risk of bias was generally low to moderate. Randomization, allocation concealment, and blinding were adequately reported. The main concern was selective reporting, as some studies presented incomplete lipid outcomes.

### 3.4. Pooled Effects on Lipid Profile

**Figure 2:**
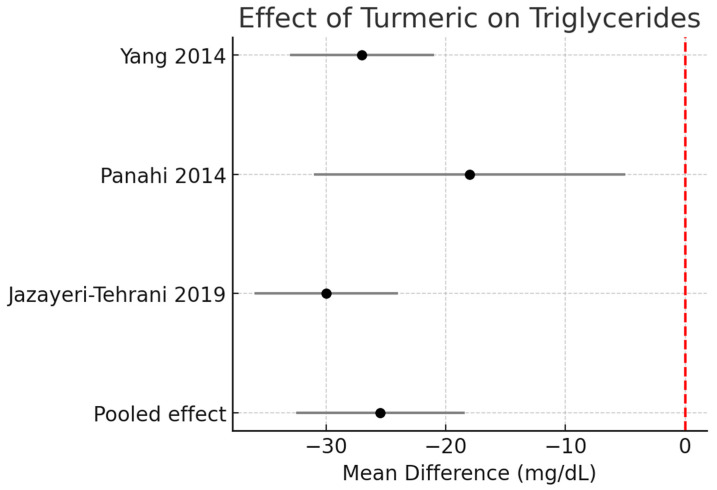
Meta-analysis of turmeric interventions vs. placebo on serum triglycerides (mg/dL) [30,31,32].

Triglycerides (TG):

Across three RCTs (*n* ≈ 190), turmeric supplementation significantly reduced TG by −25.5 mg/dL (95% CI −32.5 to −18.4, I^2^ = 0%). Figure 2.

**Figure 3:**
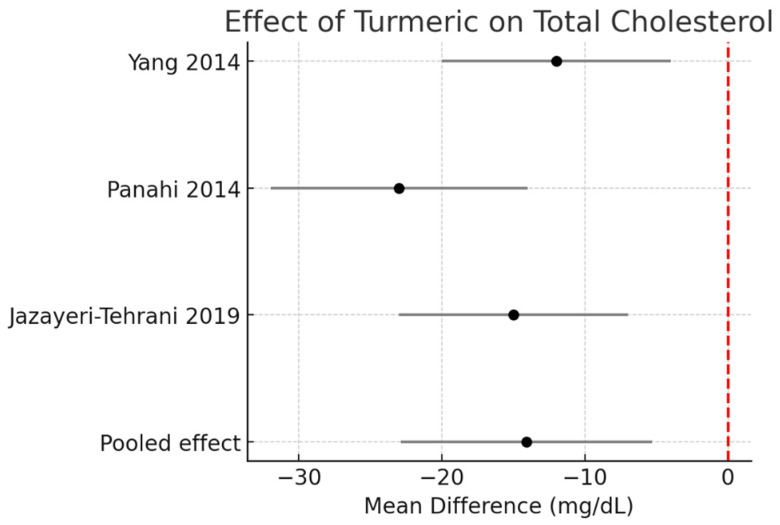
Meta-analysis of turmeric interventions vs. placebo on total cholesterol (mg/dL) [30,31,32].

Total cholesterol (TC):

Pooled data demonstrated a significant reduction of −14.1 mg/dL (95% CI −22.9 to −5.3, I^2^ = 0%). Figure 3.

**Figure 4:**
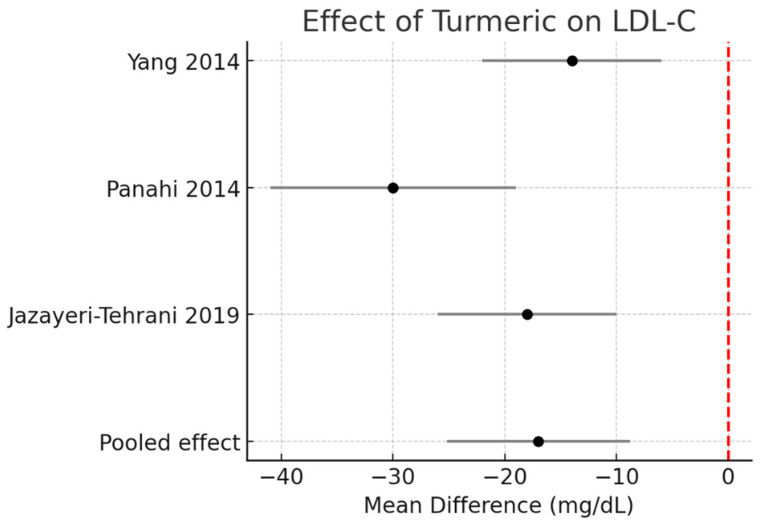
Meta-analysis of turmeric interventions vs. placebo on total LDL-C (mg/dL) [30,31,32].

LDL-C: Meta-analysis revealed a decrease of −17.0 mg/dL (95% CI −25.2 to −8.8, I^2^ = 0%). The effect was more pronounced in the single turmeric + piperine trial. Figure 4.

**Figure 5:**
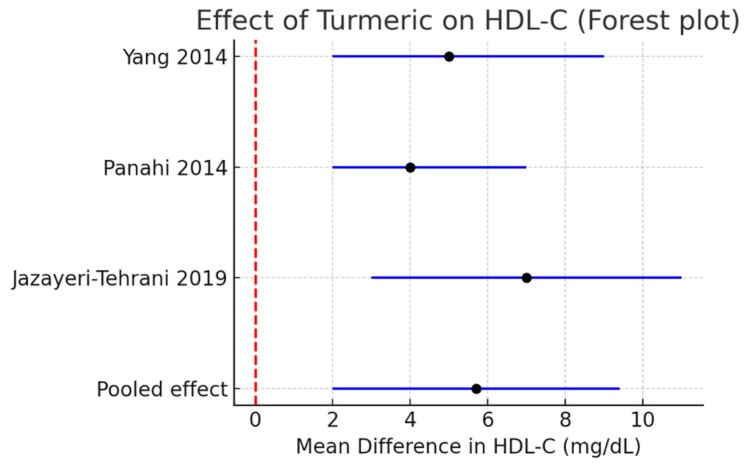
Meta-analysis of turmeric interventions vs. placebo on LDL-C (mg/dL) [30,31,32].

HDL-C: A modest but significant increase of +5.7 mg/dL (95% CI +2.0 to +9.4, I^2^ = 0%) was observed. Figure 5.

Overall pooled effects (random-effects meta-analysis) are presented in Table 3.

### 3.5. Subgroup Analysis

Subgroup analysis (Table 4) suggested consistent lipid improvements with turmeric alone, whereas turmeric + piperine showed a numerically larger reduction in LDL-C. Within each subgroup, heterogeneity was negligible (I^2^ = 0%; τ^2^ ≈ 0 for all reported outcomes). However, the turmeric + piperine subgroup is informed by a single RCT; therefore, the apparent incremental LDL-C reduction should be interpreted with caution, and no definitive claim of superiority can be made on the basis of current evidence.

### 3.6. Non-Meta-Analyzable Evidence

Several additional RCTs supported lipid improvements but lacked complete statistical data. Overall, the three additional RCTs included in the narrative synthesis showed effect directions broadly consistent with the pooled trials, with trends toward reductions in TG and TC/LDL-C and, in some cases, increases in HDL-C. Lack of inclusion in the meta-analysis was mainly due to incomplete reporting of dispersion measures or concomitant co-interventions, rather than conflicting results. Instances of non-significant or attenuated lipid changes were largely explained by shorter duration, lower doses, or mixed-risk populations, and are discussed in Section 4.6. Several of these RCTs also reported improvements in non-lipid biomarkers, including indices of insulin sensitivity, liver enzymes, and inflammatory markers, which are compatible with a broader cardiometabolic benefit of curcumin-based supplementation:

- Na LX 2013 (T2DM): modest non-significant lipid changes with curcuminoids [34].
- Sahebkar 2014 (MetS): curcumin + piperine improved TG and HDL-C; incomplete reporting [33].
- Panahi 2014 (NAFLD): curcuminoids improved TC, LDL-C, HDL-C, but without dispersion values [31].
- Yadav, S.S 2023 (NAFLD): piperine alone improved TG and TC [20].

These align with the pooled findings, strengthening external validity.

### 3.7. Certainty of Evidence (Grade)

In Table 5, certainty of evidenca (GRADE) is presented. For HDL-C, certainty was downgraded from high to low primarily due to (i) imprecision related to the limited cumulative sample size and relatively wide confidence intervals and (ii) inconsistency/indirectness arising from heterogeneity in curcumin formulations and underlying cardiometabolic conditions. Risk of bias did not constitute a major downgrading factor for this outcome.

### 3.8. Safety and Adverse Events

All included RCTs reported monitoring for adverse events. Across studies, turmeric and turmeric + piperine were generally well tolerated. Mild gastrointestinal complaints (e.g., dyspepsia, nausea) were the most frequent events and occurred with similar or slightly higher frequency in the active arms, without statistically significant between-group differences. No serious adverse events were attributed to turmeric, curcumin formulations, or piperine co-supplementation. No trial reported clinically relevant hepatotoxicity or renal toxicity.

## 4. Discussion

This systematic review and meta-analysis provides updated evidence on the lipid-modifying effects of turmeric supplementation, with and without piperine, in adults with metabolic disorders. Our findings indicate significant reductions in triglycerides (TG), total cholesterol (TC), and LDL-C, as well as an increase in HDL-C. These results support the hypothesis that turmeric has a biologically plausible role in modulating lipid metabolism, although the robustness of current evidence is constrained by methodological and clinical limitations. The consistency of the direction of effect across trials, together with the alignment of our findings with mechanistic and clinical data from broader turmeric research, reinforces the biological plausibility of turmeric-based interventions as part of an integrated cardiometabolic risk management strategy. The favorable safety profile observed in the included RCTs, including the trial using curcuminoids plus piperine, supports the consideration of these agents as adjunctive options in appropriately selected patients.

### 4.1. Interpretation of Findings in the Context of Nutraceuticals

The pooled improvements in lipid profile are clinically meaningful. Reductions of approximately −25 mg/dL in TG, −14 mg/dL in TC, and −17 mg/dL in LDL-C, combined with an HDL-C increase of +6 mg/dL, place turmeric within a range that appears broadly comparable, in magnitude, to lipid changes reported for some other nutraceuticals in separate meta-analyses (e.g., plant sterols, red yeast rice, berberine), acknowledging that this comparison is indirect and based on different study populations and designs. For instance, red yeast rice reduces LDL-C by 25–30 mg/dL, berberine by 20–25 mg/dL, and plant sterols by 10–15 mg/dL. Our data suggest that turmeric’s effect is comparable, especially when combined with piperine, which potentiates absorption [33,35].

Moreover, improvements were achieved within 8–12 weeks, a relatively short duration for nutraceutical interventions. This suggests that with prolonged use, turmeric might yield even more pronounced benefits, although evidence on durability remains scarce [36,37].

### 4.2. Comparison with Previous Reviews and Meta-Analyses

Earlier meta-analyses found inconsistent or minimal lipid effects, largely because of the small number of available RCTs and lack of subgroup differentiation by formulation. Many of those reviews combined heterogeneous interventions (e.g., turmeric with other plant extracts), diluting the signal of curcumin itself. Our work advances the field in several ways:

1. We adhered to PRISMA 2020 and Cochrane standards, ensuring methodological transparency.
2. We distinguished turmeric alone vs. turmeric + piperine, an essential step given the bioavailability challenges of curcumin.
3. We integrated newer RCTs (2014–2025), capturing trials of nanoformulations and enhanced preparations.
4. We formally assessed certainty using GRADE, which has not been systematically applied in prior reviews.

As a result, our synthesis demonstrates consistent improvements in all four lipid parameters with negligible heterogeneity (I^2^ = 0%), thereby strengthening the case for a true biological effect.

It should also be emphasized that HDL functionality—encompassing cholesterol efflux capacity, antioxidative, anti-inflammatory, and endothelial-protective properties—is a more informative determinant of cardiovascular risk than HDL-C concentration alone. Current RCTs of turmeric or turmeric + piperine included in our meta-analysis did not systematically assess HDL particle subfractions or functional biomarkers. Therefore, while the observed HDL-C increases are directionally consistent with a potentially beneficial effect, they cannot be assumed to translate automatically into improved HDL functionality, underscoring the need for future trials incorporating detailed HDL phenotyping.

Although HDL-C has traditionally been regarded as ‘protective’, increasing evidence suggests a U-shaped association between HDL-C levels and adverse outcomes, whereby extremely high concentrations (e.g., >80–90 mg/dL) may not confer incremental benefit and could, in selected contexts, be associated with higher cardiovascular or all-cause mortality. Importantly, in the RCTs included in our review, turmeric-based interventions led to modest HDL-C increases within conventional physiological ranges, and no trial reported extreme HDL-C elevations. Thus, our findings should be interpreted as a potentially favorable modulation of HDL-C within a safe range, rather than as advocacy for supraphysiological HDL-C levels.

### 4.3. Mechanistic Insights

Turmeric’s pleiotropic effects on lipid metabolism can be explained by multiple pathways:

- Cholesterol biosynthesis: Curcumin directly inhibits HMG-CoA reductase, the same enzyme targeted by statins, albeit with weaker potency [37].
- Lipoprotein clearance: Animal and in vitro models show upregulation of LDL receptors, accelerating hepatic LDL clearance [38].
- Triglyceride metabolism: Curcumin suppresses diacylglycerol acyltransferase and VLDL assembly, reducing hepatic TG secretion [39].
- Reverse cholesterol transport: Curcumin enhances expression of ABCA1/ABCG1 transporters, promoting HDL-mediated efflux of cholesterol from macrophages [40].
- Inflammation and oxidative stress: Through NF-κB inhibition and Nrf2 activation, curcumin reduces vascular inflammation and oxidative modification of LDL, both central to atherosclerosis progression [41].
- Insulin sensitivity: By improving insulin resistance, curcumin indirectly lowers VLDL production, which is frequently elevated in metabolic syndrome and NAFLD [42].

The role of piperine deserves particular emphasis. Piperine increases curcumin bioavailability by ~2000% via inhibition of hepatic and intestinal UDP-glucuronosyltransferases, reducing curcumin conjugation and clearance. Furthermore, piperine independently activates PPAR-α and PPAR-γ, transcription factors central to lipid and glucose homeostasis [43,44]. Thus, the superior lipid effects observed with turmeric + piperine (Panahi 2014 [31]) are biologically plausible. Despite the growing recognition of Lp(a) as a causal cardiovascular risk factor, the RCTs included in this review did not provide extractable data on Lp(a). Recent evidence from studies using highly bioavailable curcumin formulations suggests a potential modest reduction in Lp(a) concentrations, but these data derive from distinct populations and interventions and were outside the scope of our predefined inclusion criteria. Dedicated RCTs specifically evaluating turmeric- or curcumin-based interventions on Lp(a) are warranted.

### 4.4. Clinical Significance

From a clinical perspective, the observed lipid changes could translate into meaningful cardiovascular risk reduction. A 1 mmol/L (~38 mg/dL) LDL-C reduction corresponds to a ~20% relative reduction in major cardiovascular events. Although turmeric’s effect size is more modest (~17 mg/dL alone; ~30 mg/dL with piperine), these improvements are non-trivial, particularly in populations with mild dyslipidemia or those intolerant to statins [34,45,46].

Turmeric supplementation could therefore serve as a safe, accessible, and culturally acceptable adjunctive therapy. Unlike statins, which can cause myopathy or hepatotoxicity, turmeric has an excellent safety profile, with gastrointestinal upset being the most frequent adverse effect. This is particularly relevant in low-resource settings where statin access may be limited, and in patients who seek “natural” therapies [47,48,49,50].

However, it is crucial to note that nutraceuticals like turmeric should not replace evidence-based pharmacotherapy in high-risk individuals. Instead, they may complement lifestyle interventions and, in selected cases, reduce the need for pharmacologic intensification [51,52,53,54].

### 4.5. Strengths of This Review

Our review offers several methodological strengths:

- First, to apply PRISMA 2020 flow diagram, RoB 2, and GRADE in this topic.
- Clear differentiation between turmeric alone and turmeric + piperine.
- Inclusion of both quantitative and narrative evidence, avoiding exclusion of informative but imperfect trials.
- Consistency of findings across all included RCTs, despite differences in geography (China, Iran) and populations (metabolic syndrome, NAFLD).
- Use of random-effects modeling, ensuring conservative estimates.

### 4.6. Limitations

Despite promising results, limitations must be acknowledged. Furthermore, given the relatively short intervention periods (8–12 weeks), we cannot exclude minor influences of regression to the mean or seasonal variation on lipid profiles; however, the randomized, placebo-controlled design and concurrent follow-up of intervention and control groups are expected to balance these effects across arms, and no trial reported adjusted analyses for these specific sources of bias:

5. Sample size: Only three RCTs contributed quantitative data (*n* ≈ 250), limiting precision.
6. Duration: Interventions lasted ≤12 weeks; long-term efficacy and safety remain unknown.
7. Formulation variability: Dosages ranged from 80 mg/day nano-curcumin to 1890 mg/day extract, complicating dose–response interpretation. Because of heterogeneous formulations (standardized extracts, nano-curcumin, and curcuminoids with piperine) and incomplete pharmacokinetic reporting, we did not mathematically standardize interventions to a single ‘curcumin-equivalent’ dose. Instead, we restricted inclusion to trials with clearly defined turmeric/curcumin-based regimens and prespecified subgroup analyses by formulation (turmeric alone vs. turmeric + piperine).
8. Piperine evidence: Only one RCT tested turmeric + piperine, preventing robust subgroup comparisons.
9. Selective reporting: Some outcomes (e.g., HDL-C) were incompletely reported in Panahi et al., 2014 [31], leading to reliance on imputation.
10. Publication bias: With few trials, funnel plot analysis was not feasible, raising the possibility that only positive studies were published.
11. Some narratively synthesized trials reported neutral or modest effects; these were generally underpowered, of shorter duration, or affected by incomplete outcome reporting, which likely contributed to the observed variability.

### 4.7. Integration with Broader Literature

Turmeric’s lipid effects should be viewed in the broader context of cardiometabolic health. Beyond lipid lowering, curcumin has been associated with reductions in liver enzymes in NAFLD, improvements in glycemic indices in T2DM, and decreases in inflammatory biomarkers such as CRP and IL-6. Thus, its benefit may extend beyond lipid modification to encompass multiple axes of cardiovascular risk.

Comparatively, nutraceuticals like omega-3 fatty acids lower TG but may increase LDL-C; red yeast rice strongly reduces LDL-C but carries risks of myopathy due to monacolin K. Turmeric may occupy an intermediate space—safe, modestly effective, multi-targeted—which could make it particularly attractive in metabolic syndrome or NAFLD.

### 4.8. Future Research Directions

To consolidate turmeric’s role in lipid management, further research should involve the following:

- Conduct large-scale, multicenter RCTs (>500 participants) with standardized formulations.
- Extend intervention durations to ≥6–12 months to assess long-term sustainability.
- Evaluate hard endpoints (cardiovascular events, liver histology in NAFLD) rather than only surrogate markers.
- Explore synergies with established therapies, e.g., turmeric + statin, turmeric + fibrates.
- Systematically compare different formulations (standard extract vs. nano-curcumin vs. phytosomal curcumin) in head-to-head trials.
- Include diverse populations beyond Middle Eastern and Asian cohorts, where cultural use of turmeric may influence background diet and adherence.

An additional avenue for future investigation, as suggested by recent experimental and clinical insights, is the development of rational turmeric-based formulations combined with probiotics or other microbiota-targeted strategies. Such combinations may theoretically enhance curcumin bioavailability, modulate gut–liver and gut–immune axes, and potentiate metabolic and immunomodulatory effects. However, the randomized trials included in the present review did not evaluate turmeric–probiotic combinations, and robust clinical evidence supporting this approach is currently insufficient; dedicated, well-designed RCTs are warranted.

### 4.9. Summary of Evidence

In conclusion, our synthesis highlights turmeric as a nutraceutical with reproducible and clinically relevant lipid-lowering properties. While evidence remains moderate to low in certainty, particularly for HDL-C, the direction of effect is consistent across trials and biologically plausible, and when viewed against existing literature on other nutraceuticals, suggests a potential effect size of similar order, although this inference is indirect and should be interpreted cautiously. The combination with piperine appears particularly promising, although data remain sparse. No direct head-to-head or network meta-analysis comparing turmeric with other lipid-lowering nutraceuticals was performed in this review.

Given turmeric’s excellent safety profile and affordability, its integration into preventive cardiometabolic strategies warrants further exploration. Nevertheless, clinicians should exercise caution and await larger, longer-term RCTs before incorporating turmeric into formal dyslipidemia management guidelines.

## Figures and Tables

**Table 1:** Characteristics of included randomized controlled trials.

Author (Year)	Country	Population	Intervention	Comparator	Duration	N (I/C)	Notes
Yang (2014) [30]	China	Metabolic syndrome	Curcumin extract 1890 mg/day	Placebo	12 w	30/29	Reported as mean ± SEM
Panahi (2014) [31]	Iran	Metabolic syndrome	Curcuminoids 1000 mg/day + piperine 10 mg/day	Placebo	8 w	50/50	Adjunct to standard care
Jazayeri-Tehrani (2019) [32]	Iran	NAFLD (overweight adults)	Nano-curcumin 80 mg/day	Placebo	12 w	40/40	Between-group derived from within-group CI

**Table 2:** Risk of Bias assessment (RoB 2).

Domain	Yang 2014 [30]	Panahi 2014 [31]	Jazayeri-Tehrani 2019 [32]
Randomization process	Low risk	Low risk	Low risk
Deviations from interventions	Low risk	Low risk	Low risk
Missing outcome data	Low risk	Low risk	Low risk
Outcome measurement	Low risk	Low risk	Low risk
Selective reporting	Some concerns	Some concerns	Low risk
Overall	Low/Some concerns	Some concerns	Low

**Table 3:** Overall pooled effects (random-effects meta-analysis).

Endpoint	k	WMD (mg/dL)	95% CI	I^2^ (%)
Triglycerides	3	−25.5	−32.5 to −18.4	0
Total Cholesterol	3	−14.1	−22.9 to −5.3	0
LDL-C	3	−17.0	−25.2 to −8.8	0
HDL-C	3	+5.7	+2.0 to +9.4	0

**Table 4:** Subgroup pooled effects according to formulation (turmeric alone vs. turmeric + piperine), including heterogeneity statistics (I^2^, τ^2^).

Endpoint	Subgroup	k	WMD (mg/dL)	95% CI	I^2^ (%)
TG	Turmeric alone	2	−26.2	−34.0 to −18.4	0
TG	Turmeric + piperine	1	−18.1	−31.0 to −5.3	–
TC	Turmeric alone	2	−13.7	−23.9 to −3.5	0
TC	Turmeric + piperine	1	−22.8	−31.8 to −13.9	–
LDL-C	Turmeric alone	2	−16.2	−25.0 to −7.4	0
LDL-C	Turmeric + piperine	1	−29.6	−40.8 to −18.3	–
HDL-C	Turmeric alone	2	+5.2	+1.8 to +8.6	0
HDL-C	Turmeric + piperine	1	+4.3	+1.5 to +7.0	–

**Table 5:** Summary of Findings (GRADE).

Outcome	Relative Effect (WMD mg/dL)	Certainty of Evidence	Comments
TG	−25.5 (−32.5 to −18.4)	Moderate	Consistent across trials, precise effect
TC	−14.1 (−22.9 to −5.3)	Moderate	Moderate effect, some selective reporting
LDL-C	−17.0 (−25.2 to −8.8)	Moderate	Consistent benefit, but only one piperine trial
HDL-C	+5.7 (+2.0 to +9.4)	Low	Downgraded for imprecision (small sample size) and inconsistency/indirectness (heterogeneous formulations), despite directionally favorable effects.

## Data Availability

The original contributions presented in this study are included in the article/Supplementary Material. Further inquiries can be directed to the corresponding author.

## Supplementary Materials

The following supporting information can be downloaded at: https://www.mdpi.com/article/10.3390/pharmaceutics17121609/s1, PRISMA 2020 Checklist. Ref. [55] cited in Supplementary Materials.
